# Unraveling the cytotoxic potential of Temozolomide loaded into PLGA nanoparticles

**DOI:** 10.1186/2008-2231-22-18

**Published:** 2014-01-10

**Authors:** Darshana S Jain, Rajani B Athawale, Amrita N Bajaj, Shruti S Shrikhande, Peeyush N Goel, Yuvraj Nikam, Rajiv P Gude

**Affiliations:** 1 Department of Pharmaceutics, C.U. Shah College of Pharmacy, SNDT Women’s University, Juhu Tara Road, Santacruz (West), Mumbai 400 049, India; 2 SVKM’s Dr. Bhanuben Nanavati College of Pharmacy, Vileparle, Mumbai 400 056, India; 3 Gude Lab, Advanced Centre for Treatment, Research & Education in Cancer (ACTREC), Tata Memorial Centre, Kharghar, Navi Mumbai 410 210, India

**Keywords:** C6 cell line, Gliomas, PLGA nanoparticles, Temozolomide

## Abstract

**Background:**

Nanotechnology has received great attention since a decade for the treatment of different varieties of cancer. However, there is a limited data available on the cytotoxic potential of Temozolomide (TMZ) formulations. In the current research work, an attempt has been made to understand the anti-metastatic effect of the drug after loading into PLGA nanoparticles against C6 glioma cells.

Nanoparticles were prepared using solvent diffusion method and were characterized for size and morphology. Diffusion of the drug from the nanoparticles was studied by dialysis method. The designed nanoparticles were also assessed for cellular uptake using confocal microscopy and flow cytometry.

**Results:**

PLGA nanoparticles caused a sustained release of the drug and showed a higher cellular uptake. The drug formulations also affected the cellular proliferation and motility.

**Conclusion:**

PLGA coated nanoparticles prolong the activity of the loaded drug while retaining the anti-metastatic activity.

## Background

Glioblastoma multiforme is WHO classified grade IV type of brain tumors depicting pleomorphism and atypical nuclei [1]. The current therapeutic modality for the treatment includes surgery followed by chemotherapy and radiation. Despite the progress in understanding of molecular basis in the gliomas, the prognosis of tumors remains dismal [2]. The main reason for the poor prognosis is the complexity of the brain and presence of Blood Brain Barrier (BBB). This comprises of endothelial cells that does not allow entry of exogenous material, bacteria, viruses and chemotherapeutic agents. Further, the expression of the efflux transporters adds to its complexity [3].

However, BBB allows the passage of small sized particles that are hydrophobic in nature [4]. Nanoparticles viz. solid lipid nanoparticles, polymeric nanoparticles, nano emulsions have been fabricated and used against gliomas [5-7]. Small sized particles are better permeated though the barrier with the target ability to the cancerous cells. TMZ has been a drug of choice and is used as a first line agent for the treatment of gliomas after its surgical resection. However, due to a very short half life of 1.8 h and protein binding of 15% [8,9] repeated administration of the drug is required. In addition, less site specificity is demonstrated by the drug and thus the amount of the drug reaching the tumor site becomes limited. The probability of aiming the cancerous cells with sustained release of the loaded drugs/agents by nanoparticles prompted us to undertake the present work. Nanoparticles loaded with doxorubicin, daunorubicin, epirubicin, temozolomide, methotrexate and many other such chemotherapeutic agents have been prepared and characterized for various physico-chemical properties [10-14].

Polymeric nanoparticles are amongst the most preferred delivery system for treatment of cancers due to higher penetrability, sustainability, degradability and better payload. The reason for selecting PLGA is based on its tunable properties and biodegradability. Biodegradability leads to formation of lactic acid and glycolic acid that are readily cleared form the body as by products [15]. There are no reports for the *in vitro* performance of TMZ encapsulated in nanoparticulate formulations till date.

In this purview, we had decided to fabricate PLGA nanoparticles loaded with anti cancer agent TMZ for targeting glial cells. The prepared nanoparticles could thus reach the target cells by passage though the BBB and demonstrate better penetrability though the cancerous cells. Further, we have evaluated the performance index of both the free and encapsulated drug. The results clearly suggest the applicability of PLGA coated nanoparticles for the management of gliomas.

## Methods

### Cell lines

The C6 glioma cell line was procured from NCCS, Pune, India. It was maintained in HAM’s 12 medium (GIBCO) supplemented with 10% heat inactivated fetal bovine serum, FBS (GIBCO) and primocin (100 μg/mL). Cultures were maintained at 37°C in 5% CO_2_ humidified atmosphere.

### Materials

Temozolomide was a generous gift sample from Cipla Pvt. Ltd. Vikhroli, Mumbai. PLGA was obtained as a gift sample from PURAC (PDLG 50:50 grade). Poloxamer 188 (Lutrol F 68) was obtained as a gift sample from BASF Ltd. Mumbai. All the other chemicals/reagents used were either of analytical grade or the highest purity commercially available.

### Preparation of nanoparticles

PLGA nanoparticles were prepared by a method principally involving solvent diffusion technique [15]. Briefly, 50 mg of PLGA was solubilised in 4 mL of acetonitrile. To the same solution 30 mg of the drug was added and solubilised. The above solution was emulsified with an aqueous solution (4 mL) comprising of 0.5% poloxamer 188 using IKA -18 Ultraturrax. Emulsified organic solvent was carefully poured into 8 mL of prechilled 0.5% poloxamer 188 solution kept stirring over a magnetic stirrer. Solvent was allowed to slowly diffuse leaving behind homogenous nanoparticles. Particle size and zeta potential for the developed particles were noted using Malvern Zetasizer 90S.

### Particle size analysis and polydispersity index measurements

Particle size and polydispersity index for the developed nanoparticles were noted using Malveren Zetasizer instrument 90S (Ver 6.12.). Particle size indicates the average size acquired by the nanoparticles when dispersed in water whereas the polydispersity index depicted the homogeneity in distribution of these particles. In our study, 20 μL of the nanoparticulate suspension was suitably diluted with HPLC grade water in the polystyrene cuvette. The cuvette was then placed in the pathway of scattered light to record the fluctuations in the intensity of the light due to the presence of the particles. The intensity of the scattered light was measured at 90° using Malvern software to give the hydrodynamic diameter and the polydispersity index [16].

### Zeta potential and diffusion coefficient measurements

Zeta potential measurements enable to determine surface charge acquired by the particles in solution. Samples were diluted using the procedure discussed for particle size measurement. A dip cell with electrode was inserted into the cuvette and then placed in Malvern Zetasizer 90 S. Diffusion coefficients and the particle mobility was noted for each sample. An average of approximately 15 runs was measured at 90° at the stationery level in the cylindrical cell. Auto correlated software depicted the zeta potential values for each sample depending upon the mobility of the particles towards the electrode.

### *In- vitro* dissolution study

**Figure 1:**
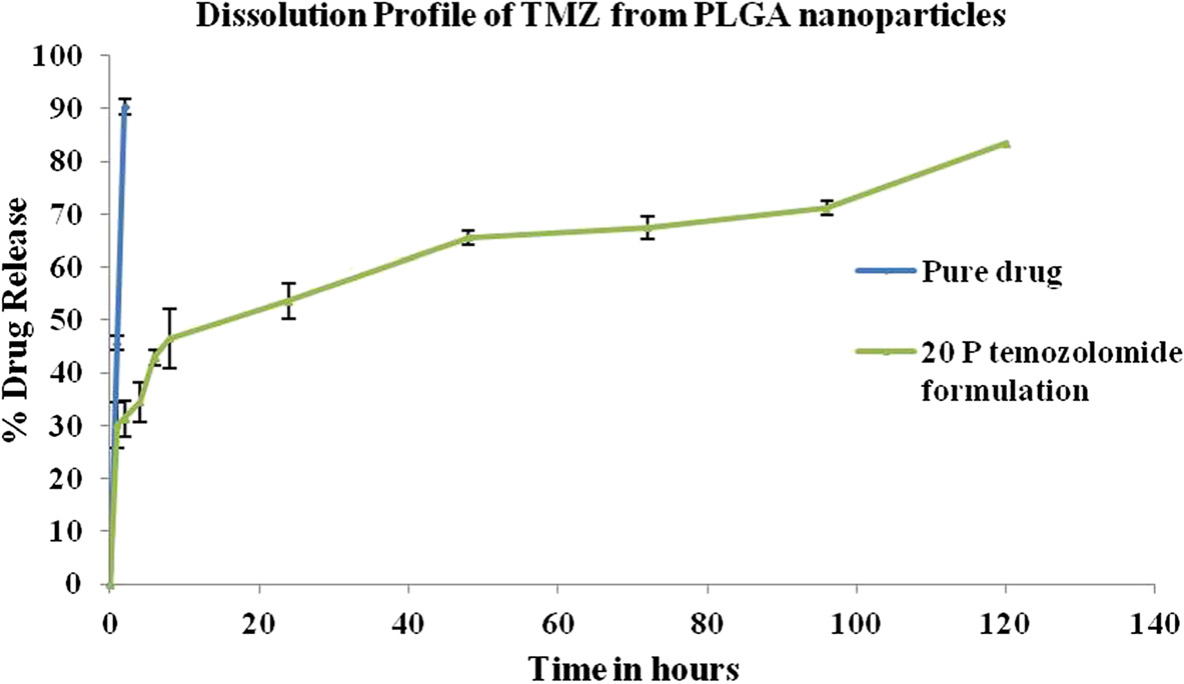
***In vitro *Dissolution profile of pure drug and TMZ- PLGA nanoparticles.** An immediate release of 30% fraction of drug (unentrapped drug) in initial 2 h is seen, followed by sustained release of drug up to 120 h indicating the biphasic release pattern for the drug which is typical of PLGA nanoparticles.

Diffusion though dialysis membrane was assessed to determine the diffusion and further dissolution of the drug in the dissolution medium. Quantification of the drug from the withdrawn aliquots during *in-vitro* dissolution was performed using developed and prevalidated UV method (JASCO). Pure Drug and nanoparticle batches equivalent to 20 mg of drug were weighed and transferred to dialysis membrane {cut off 6-8 KDa and diameter of 21.5 mm (Hi media dialysis membrane no. 50) sealed at one end by thread. Approximately, 1 mL of dissolution medium (sodium acetate buffer pH 5) was added in these bags, and another end was sealed. Prepared dialysis bags were dialyzed by suspending in 500 mL of pH 5 sodium acetate buffer placed in dissolution flask (Apparatus: Dissolution tester: Electrolab 6 T). Dissolution was performed in pH 5 sodium acetate buffer dissolution medium at rotation speed of 100 rpm. Intermittently aliquots were withdrawn at prefixed time intervals. Each time 1 mL was withdrawn and replenished with the same amount of dissolution medium during the study. Volume of the aliquots was made to 10 mL with dissolution medium and absorbance was measured at λmax of 255 nm on a UV spectrophotometer. Calculation for % drug release was performed at each time point and graphs indicating % drug released vs. time were plotted and are represented in Figure 1.

### Cellular proliferation using MTT cytotoxicity assay

**Figure 2:**
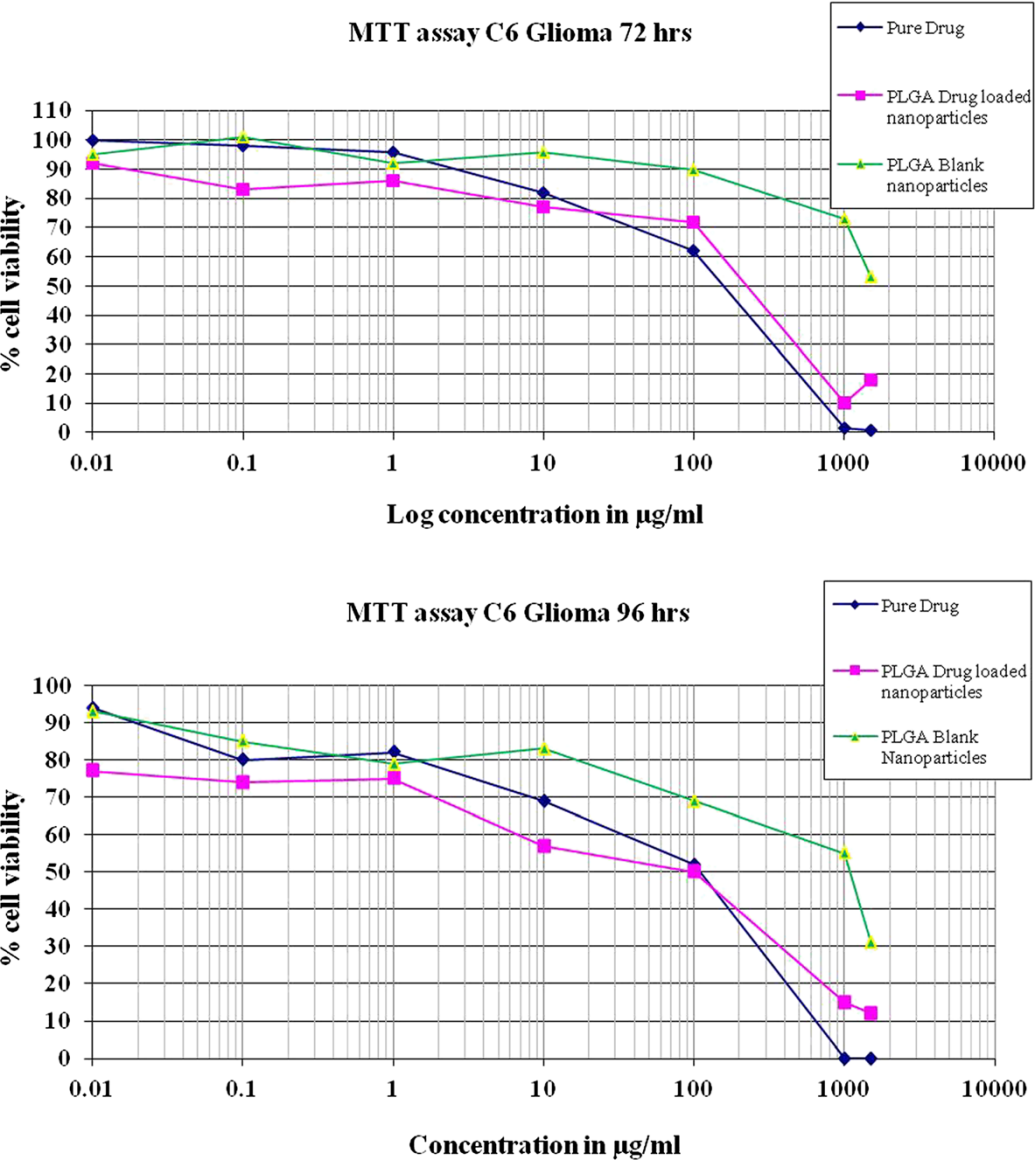
**MTT assay performed for the pure drug and drug loaded PLGA nanoparticles.** The observed IC_50_ was 150 μg/mL TMZ in pure form on C6 glioma irrespective of the time. However, the same drug when loaded into nanoparticles demonstrated an IC_50_ of 200 μg/mL and 150 μg/mL after 72 h and 96 h, respectively.

Cytotoxicity was determined using MTT assay as previously reported [17,18]. Briefly, 2500 and 1500 cells/well were seeded in a 96-well plate respectively. After 24 h cells were treated with pure drug TMZ, PLGA blank nanoparticles and PLGA nanoparticles loaded with TMZ at varying doses of (1,500-0.01 μg/mL). Plates were then incubated at 37°C for 72 h and 96 h in CO_2_ incubator. The drug was discarded and the wells were washed twice with PBS at respective time points. MTT (1 mg/mL) was added to all the wells and incubated overnight. The plates were later centrifuged and the supernatant was finally discarded. The formazon crystals formed were dissolved in DMSO and the readings were taken using ELISA plate reader at dual wavelengths of 540/690 nm respectively. IC_50_, the concentration required to kill 50% of the cells by TMZ was calculated. The graph was plotted on a logarithmic scale as percentage viability versus drug concentration and is represented in Figure 2.

### Colony formation assay

**Figure 3:**
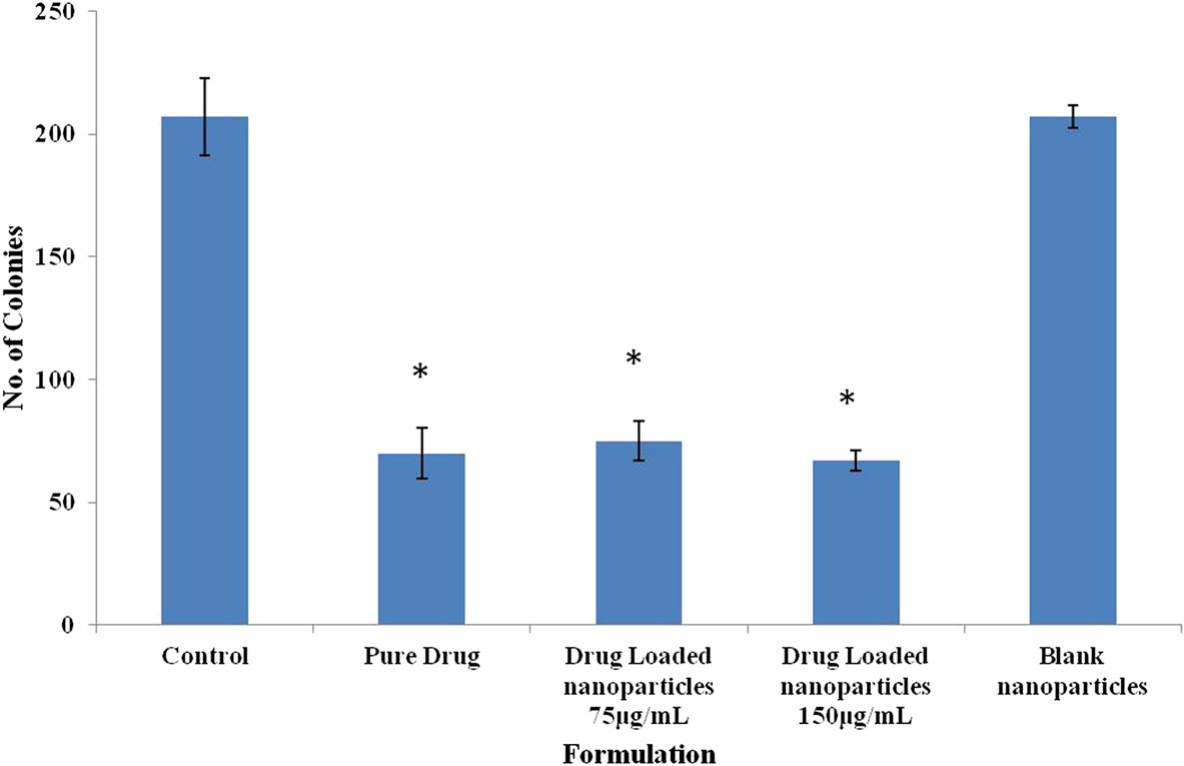
**Clonogenic assay for pure drug, TMZ loaded PLGA nanoparticle formulations and placebo PLGA nanoparticles.** The numbers of colonies for untreated and pure drug treated cells were found to be 221.00 ± 15.56 and 67.00 ± 10.26 respectively. However, in case of drug loaded nanoparticles the number of colonies were 75.00 ± 8.00, 70.66 ± 4.00 at concentrations of 75 μg/mL and 150 μg/mL respectively. (*P < 0.05).

Clonogenic assay was done to assess the long term cytotoxicity of different formulations. 600 cells were seeded in a 35 mm plate and after stabilization [19] treated with developed formulations: PLGA formulations (Drug loaded and blank) and pure drug. These were removed after an incubation period of 24 h, and the plates were washed with PBS to remove traces of the drug. Cells were later incubated in the presence of complete media for a period of 8–10 days. Fixation was done using 70% chilled methanol followed by staining using 1% crystal violet. Colonies containing 50 or more cells were counted and the results were plotted as number of colonies versus drug/formulation (Figure 3).

### Wound scratch assay

**Figure 4:**
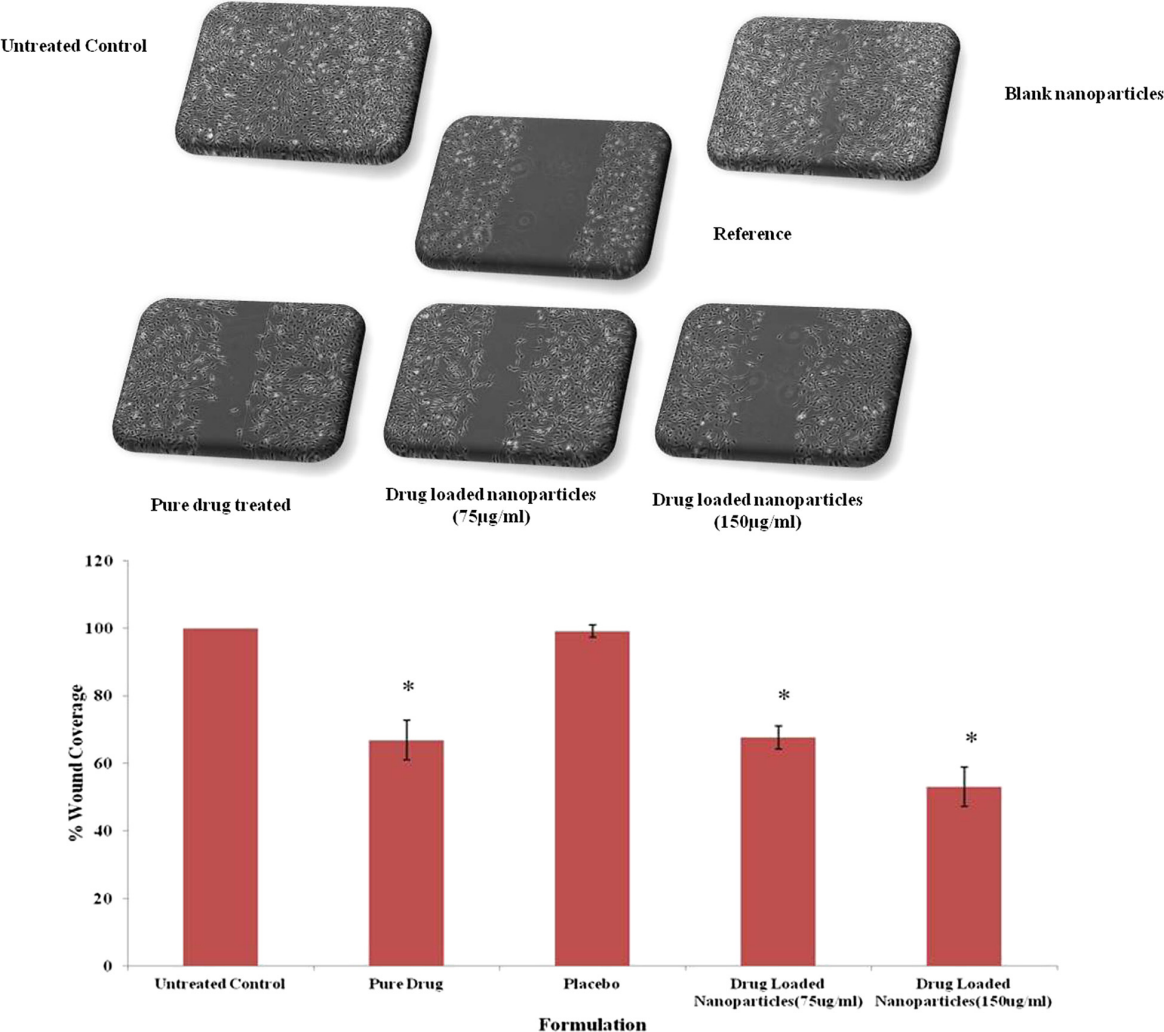
**Wound Scratch assay to assess the role of developed nanoformulations on cellular motility.** The wound coverage was found to be 66.84 ± 5.81, 67.59 ± 3.39 for TMZ and loaded nanoparticles at 75 μg/mL dose compared to the control (100%). A significant decrease in wound coverage at 150 μg/mL of nanoparticles concentration i.e 53.00 ± 5.82 is observed. However, no significant difference between placebo and control groups was observed (*P < 0.05).

Wound scratch assay was performed as per the earlier reports [18,20,21]. Approximately, 0.6 million cells were seeded into 35 mm plates. On the following day, cells were treated with 2 μg/mL mitomycin C for 1 h. The cells in the centre of the plates were later scraped using a sterile tip so as to form a wound. Sub-toxic doses of TMZ (75 μg/mL and 150 μg/mL), TMZ loaded formulations and PLGA formulations (equivalent to pure drug) were then added and incubated for 24 h. Drug formulations were removed and the plates were fixed using 70% methanol. The wound width was measured using the AxioVision Rel 4.8 imaging software, and the results were plotted as percent wound closure with respect to control. The controls were considered to be covered 100%. Results are represented in Figure 4.

### Cellular morphology in presence of TMZ loaded in nanoparticulate formulation

**Figure 5:**
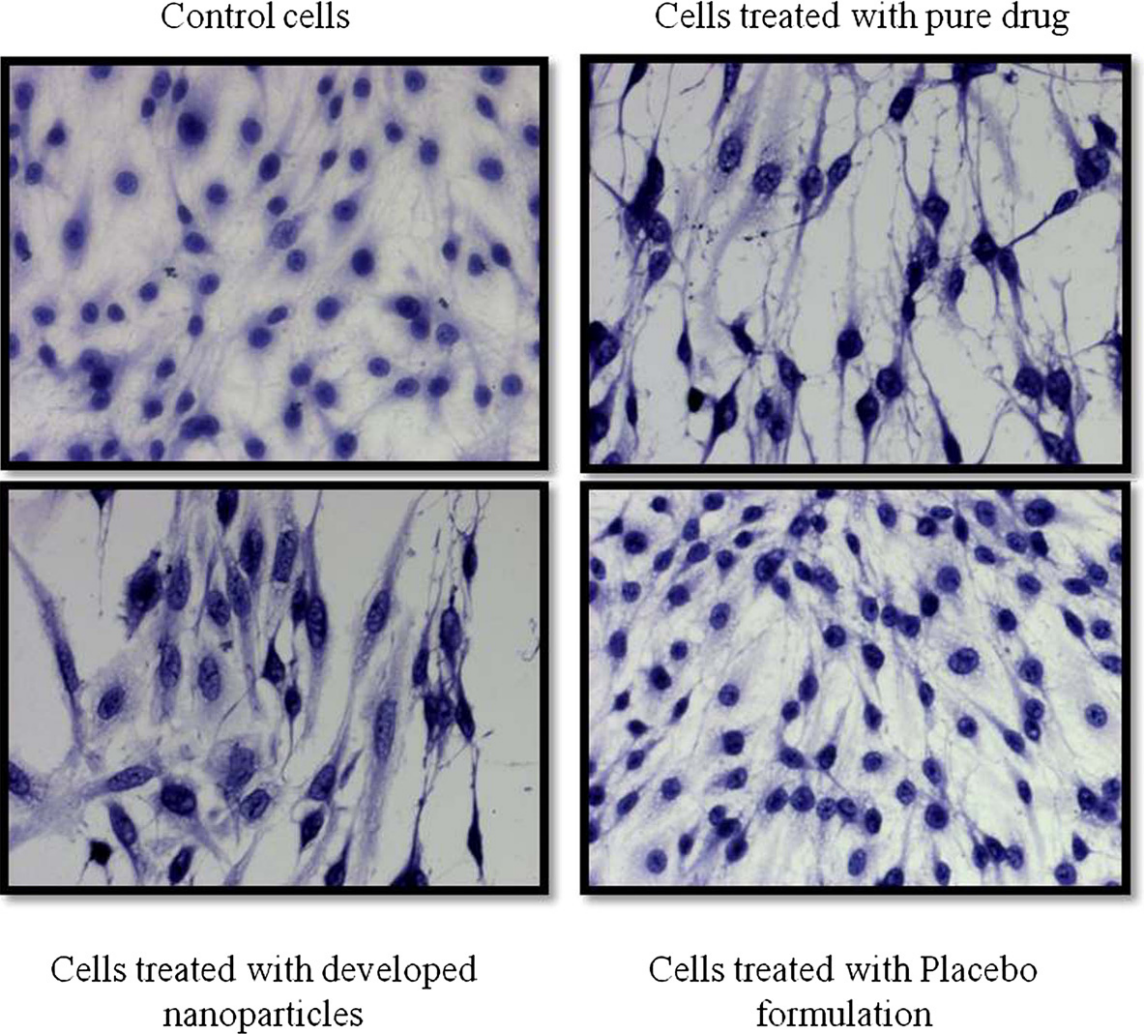
**Changes in cellular morphology using HE staining.** The spindle shape cells of C6 glioma is distorted after treatment with free TMZ and TMZ loaded formulations. Further, stress on the spindle fibres with further rounding up of the cells is being observed.

Haematoxylin-Eosin (HE) staining was performed to observe the morphological changes upon drug treatment as described previously [17,18]. Sub-confluent cells were allowed to be grown on coverslips. Cells were then treated with TMZ, PLGA blank formulations and TMZ loaded into nanoparticulate formulations (concentration equivalent to 75 μg/mL) as done earlier and fixed using 70% chilled methanol. The cover slips were then stained using haematoxylin and eosin subsequently. Mounting was done on the glass slides using DPX mountant. Cells were later observed under Zeiss upright microscope. The results are shown in Figure 5.

### Cellular uptake using confocal microscopy

**Figure 6:**
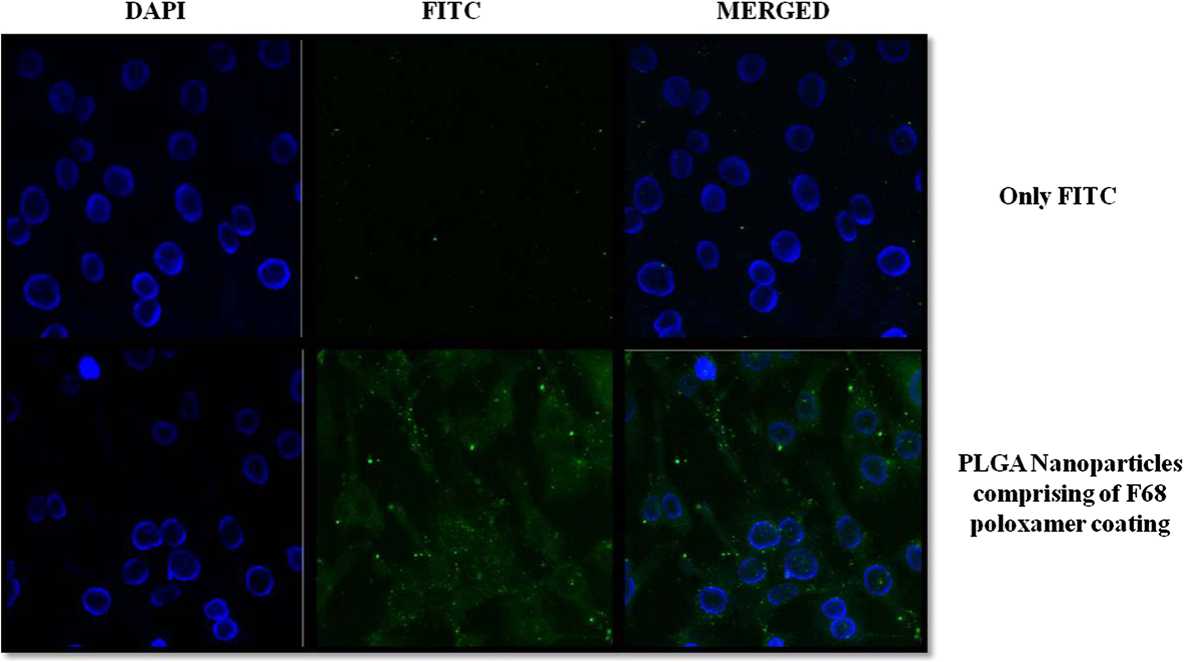
**Immunofluorescence studies for cellular uptake of developed nanoparticles.** Cells treated with FITC showed almost negligible fluorescence indicating absence of its uptake. However, cells treated with FITC loaded nanoparticles exhibited strong green fluorescence. Nucleus is stained with DAPI represented in dark blue colour.

The uptake of nanoparticles was performed using particles loaded with FITC, a hydrophilic dye. Sub-confluent cultures of C6 glioma were treated with only FITC and FITC loaded nanoparticles for 2 h respectively. Cells were later washed with PBS and fixed using 1% paraformaldehyde (PFA). The cells were then treated with DAPI and then washing was done thrice with PBS. Mounting of coverslips was done using 2.5% DABCO on glass slides and then sealed with nail paint. Acquisition was done on LSM 510 confocal microscope from Zeiss at 63X. LSM image browser software was used for data analysis (Figure 6).

### Cellular uptake using flow cytometry

**Figure 7:**
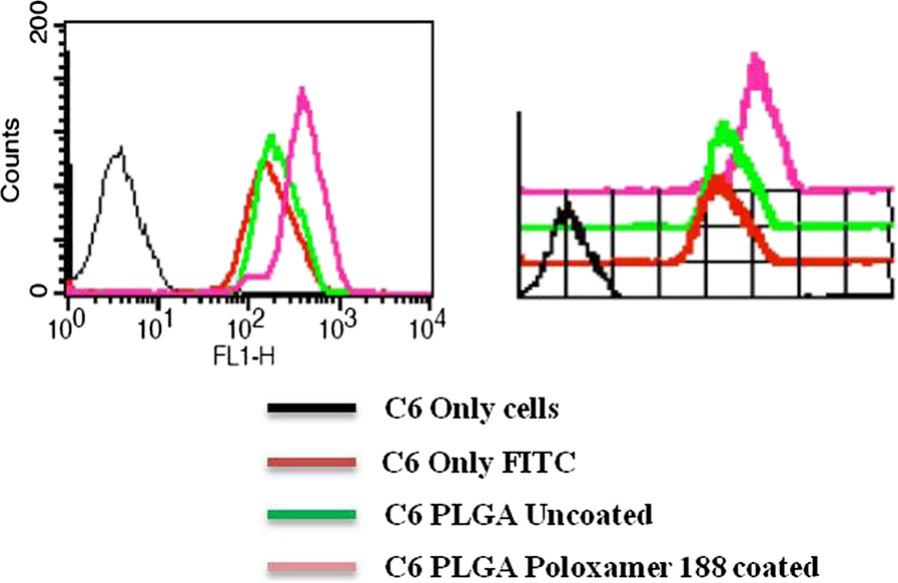
**Cellular uptake using flow cytometry.** Uptake of PLGA-poloxamer coated nanoparticles is higher (pink color) than the uncoated nanoparticles (green color) and cell treated with only FITC (red color). Black colour indicates background fluorescence because of only cells. Both 2D and 3D representations are shown.

Flow cytometric analysis was done to confirm our previous findings. Sub-confluent cultures of C6 glioma were treated with FITC and its loaded nanoparticles as done earlier [22]. Cells were thereafter harvested and fixed using 1% PFA. Cells were then washed using PBS and later suspended in the same. Acquisition was done using FACS Calibur and the results were analysed by CellQuest software (Figure 7).

### Statistical analysis

All the experiments had been performed at least thrice. The results are indicated as Mean ± SD. One way ANOVA was used for the purpose of statistical significance. The results were considered to be statistically significant where *P < 0.05.

## Results and discussion

### Fabrication of nanoparticles

Solvent diffusion method was used for the fabrication of nanoparticles. Developed nanoparticles were subjected to particle size and zeta potential assessment. The particle size for the pure drug was found to be 1256 ± 82 nm (Approximately 1.3 μm crystalline structure particles). A size range of 150-160 nm was found for the developed nanoparticles with poly dispersity index values suggesting that the developed nanoparticles were monodisperse. The developed nanoparticles were then lyophilized and the assessment of the size was performed post lyophilisation. The developed lyophilized nanoparticulate formulation maintained the particle size even after reconstitution indicating stability and robustness of the developed nanoparticles. Zeta potential for the pure drug was found to be 17.40 ± 0.256 mV and for the developed nanoparticles was found to be - 20.50 ± 0.069 mV.

There are numerous methods to design PLGA nanoparticles viz. solvent evaporation, microfluidisation, solvent diffusion, nanoprecipitation and salting out. The designed nanoparticles were developed successfully with solvent diffusion technique with acetonitrile as organic solvent. As the particles were designed for treatment of glioblastoma multiforme, the major obstacle to the delivery of particles would be passage though the BBB. However, BBB permit passage of small size entities (below 200 nm) which are hydrophobic in nature and hence particle size is an important parameter for entities aiming to pass the blood brain barrier. As the particle size range of our designed nanoparticles was below 200 nm better permeation can be expected though the barrier. The surface charge on the particle could be determined by the zeta potential. Negative values for the pure drug indicate that the charge on the surface is negative. The negatively charged drug particles are taken up the liver and further phagocytosized, thus only a small fraction of the drug remains in the circulation [23,24]. With the designed nanoparticles negative charge of the particles was maintained with the readings at the higher end of the negative charge. Higher values of the zeta potential determine the stability of the particle. Further, transmission electron microscopy images depicted formation of roughly round PLGA drug loaded nanoparticles. Poloxamer 188 layer with the thickness of around 6-10 nm was found using transmission electron microscopy [15].

### *In vitro* dissolution profile

Dissolution was performed in sodium acetate buffer pH 5, considering the stability of the drug. TMZ is unstable at physiological pH and rapidly undergoes hydrolysis to form MTIC and AIC as metabolites. Pure drug diffuses out of the dialysis bag within 2 h as indicated in the graph plotted for TMZ. This reading can be correlated with the half life of the drug being 1.8 h that means the drug is readily available for its action *in vivo*. However, the developed nanoparticles demonstrated a slow release pattern of the drug when loaded into PLGA nanoparticles. The half life of the drug is extended as indicated from dissolution profile. An immediate release of 30% fraction of drug (unentrapped drug) in initial 2 h is seen, followed by sustained release of drug up to 120 h indicating the biphasic release pattern for the drug which is typical of PLGA nanoparticles. The developed nanoparticles could sustain the drug release and extend the half life of drug. The data warrants the avoidance of repeated administration of TMZ and this would be of clinical significance because the developed formulation avoids repeated administration of the active component *in vivo*. This action was further confirmed and correlated with performed *in vitro* cell line study.

### Nanoparticles exert anti-proliferative effects in a dose and time dependent manner

Time and dose-dependent based MTT studies were performed to determine the IC_50_ of TMZ (72 h and 96 h). The observed IC_50_ for pure TMZ was found out to be 150 μg/mL against C6 glioma irrespective of the time. However, the same drug when loaded into nanoparticles demonstrated an IC_50_ of 200 μg/mL and 150 μg/mL after 72 h and 96 h, respectively. As the time of exposure of formulations to the cells was increased, IC_50_ showed a significant decrease. This further correlated with the dose. As the dose of the pure drug and drug loaded formulation was increased IC_50_ of the compound decreased. However, for pure drug IC_50_ was concentration but not time dependent. The developed formulations released the drug at a very slow rate and hence a small proportion of drug is available to exhibit action each time (drug release profile in Figure 2). The obtained results can be correlated with the dissolution profile of the drug. The diffusion of the drug from the formulation continued for more than 120 h. Hence, even after 96 h IC_50_ for pure drug and drug loaded nanoparticles did not match, confirming slow release of the drug from the developed formulation, as well as time and concentration dependent cytotoxicity of developed formulations. Further, to confirm the absence of cytotoxic action of the excipients used in the developed formulation, MTT assay was also performed for the blank formulation. High IC_50_ (> 1000 μg/mL after 96 h exposure) value of the blank formulation depicts the safety of the polymer and other excipients used for fabricating the nanoparticles. Based on these observations, 75 μg/mL and 150 μg/mL of TMZ in free and loaded form were selected for performing the subsequent experiments with 24 h exposure.

### Effect of nanoparticles on the clonogenic potential

Anti-proliferative activity of the cells was performed using clonogenic assay. The numbers of colonies for untreated and pure drug treated cells were found to be 221.00 ± 15.56 and 67.00 ± 10.26. The number of colonies for drug loaded nanoparticles was found to be 75.00 ± 80, 70.66 ± 40 at concentrations of 75 μg/mL and 150 μg/mL respectively (*P* < 0.05). Cancer cells show a self sufficiency towards growth signals [25]. These results indicate that the cells lose the ability to replicate in the presence of drug and its formulations. The possible mechanism might be due to inhibition of EGFR and MAPK pathways by TMZ as reported earlier, that play key roles in the process of proliferation [26,27].

### Nanoparticles affect motility of C6 glioma cells

Both pure TMZ and its nanoparticles formulations at dosage of 75 μg/mL showed a similar reduction in motility. The wound coverage was found out to be 66.84 ± 5.81, 67.59 ± 3.39 for pure and nanoparticles at 75 μg/mL dose compared to the control (100%). However, there was a significant decrease in wound coverage at 150 μg/mL of nanoparticles concentration i.e 53.00 ± 5.82 (*P* < 0.05). There was no significant difference between placebo and control groups respectively. The differences in migration between pure drug and drug loaded nanoparticles formulation (150 μg/mL) can be attributed to the slower diffusion of the drug from the nanoparticles. The effect of TMZ on Rho GTPase signaling might be the possible reason for hindering migration as earlier reported [27].

### Changes in cellular morphology upon TMZ and PLGA nanoparticles treatment

C6 glioma cells exhibits atypical nuclei with pleomorphism characteristic of human glioma cancer. The spindle shape cells of C6 glioma is distorted after treatment with free TMZ and TMZ loaded formulation. Stress on the spindle fibres with further rounding up of the cells is being observed. Further, there is a decrease in cell volume and number with both the regimens, demonstrating equal efficacy of the developed drug loaded nanoparticles.

### Immuno fluorescence showed a higher uptake of developed nanoparticles

C6 glioma cells were treated with FITC loaded into PLGA nanoparticles. Images for the uptake are depicted in Figure 6. Cells treated with pure dye showed almost negligible or background fluorescence indicating no uptake of the dye by the cells. However, cells treated with FITC loaded nanoparticles exhibited strong green fluorescence. The nucleus of the cells is stained with DAPI represented in blue colour. These results indicated that the developed nanoparticles are able to modulate the uptake of hydrophilic dye after its encapsulation in the particle. PLGA being a hydrophobic polymer with tunable properties masks the entity of FITC, a hydrophilic agent not readily permeating into the cells. Thus, it can be concluded that the uptake of hydrophilic/amphipathic agent will be enhanced by using PLGA designed nanoparticles.

### Flow cytometric analysis showed an increase in uptake of developed nanoparticles

To further corroborate our earlier findings using confocal microscopy, we had performed the cellular uptake of the developed nano formulations in C6 glioma cells by flow cytometry. As can be seen in Figure 7, uptake of PLGA-poloxomer is high (pink color) than the uncoated (green color) and cell treated with only FITC (red color). These observations further substantiate our earlier findings.

## Conclusions

The present study was performed to develop effective formulations targeting across the BBB. TMZ loaded nanoparticles were successfully prepared using solvent evaporation method. The developed nanoparticles exhibited slow release of the drug with prolongation of its half life. Further, cellular uptake of the nanoparticles was enhanced as confirmed using confocal and flow cytometry. These properties certainly enhance the safety profile of the drug with reducing its toxicity. Hence, it may be concluded Poly Lactic glycolic acid (PLGA) nanoparticles sustain the cytotoxic action of TMZ in C6 Glioma cells.

## Competing interests

The authors declare that they have no competing interests.

## Authors’ contributions

DJ collected data, performed experiments such as fabrication of nanoparticles and manuscript writing. RA, AB also helped in writing the manuscript and correcting the manuscript. SS helped in collecting the information and data related to nanoparticulate formulations. PG performed uptake studies, drafting manuscript and performing cell line experiments along with YN. RG is project collaborator and provided technical expertise in correcting and drafting the manuscript. All authors read and approved the final manuscript.
